# Unscheduled-Return-Visits after an Emergency Department (ED) Attendance and Clinical Link between Both Visits in Patients Aged 75 Years and Over: A Prospective Observational Study

**DOI:** 10.1371/journal.pone.0123803

**Published:** 2015-04-08

**Authors:** Laurent Pereira, Christophe Choquet, Anne Perozziello, Mathias Wargon, Gaelle Juillien, Luisa Colosi, Romain Hellmann, Michel Ranaivoson, Enrique Casalino

**Affiliations:** 1 Assistance Publique-Hôpitaux de Paris (AP-HP), University Hospital Bichat-Claude Bernard, Emergency Department, Paris, France; 2 Study Group for Efficiency and Quality of Emergency Departments and Non-Scheduled Activities Departments, Paris, France; 3 Medical Information Systems Program (PMSI), University Hospital Bichat-Claude Bernard, Paris, France; 4 Hôpital Saint Camille, Bry sur Marne, France; 5 EA 7334 REMES, Université Paris Diderot, Sorbonne Paris Cité, Paris, France; Waseda University, JAPAN

## Abstract

**Background:**

Predictors of unscheduled return visits (URV), best time-frame to evaluate URV rate and clinical relationship between both visits have not yet been determined for the elderly following an ED visit.

**Methods:**

We conducted a prospective-observational study including 11,521 patients aged ≥75-years and discharged from ED (5,368 patients (53.5%)) or hospitalized after ED visit (6,153 patients). Logistic Regression and time-to-failure analyses including Cox proportional model were performed.

**Results:**

Mean time to URV was 17 days; 72-hour, 30-day and 90-day URV rates were 1.8%, 6.1% and 10% respectively. Multivariate analysis indicates that care-pathway and final disposition decisions were significantly associated with a 30-day URV. Thus, we evaluated predictors of 30-day URV rates among non-admitted and hospitalized patient groups. By using the Cox model we found that, for non-admitted patients, triage acuity and diagnostic category and, for hospitalized patients, that visit time (day, night) and diagnostic categories were significant predictors (p<0.001). For URV, we found that 25% were due to closely related-clinical conditions. Time lapses between both visits constituted the strongest predictor of closely related-clinical conditions.

**Conclusion:**

Our study shows that a decision of non-admission in emergency departments is linked with an accrued risk of URV, and that some diagnostic categories are also related for non-admitted and hospitalized subjects alike. Our study also demonstrates that the best time frame to evaluate the URV rate after an ED visit is 30 days, because this is the time period during which most URVs and cases with close clinical relationships between two visits are concentrated. Our results suggest that URV can be used as an indicator or quality.

## Background

Demographic data indicate a sharp increase in the number of elderly individuals [1]. The number of visits to Emergency Departments (ED) by elderly patients is likely to increase over the coming years [2], as is the number of admissions from these EDs [3]. Improving and reorganizing elderly care has become a healthcare priority, [4] since older patients are more likely to experience higher rates of adverse events [5, 6]. Older people make up a large proportion of ED patients and increasing age accounts for 18% of the observed reduced performance in EDs measured between 1994 and 2004 [7, 8, 9].

Readmissions and unscheduled return visits to the ED (URV) are associated with increased mortality and excessive hospital costs [10]. The rate of readmission after hospital discharge has been chosen as a measure of healthcare quality [11–13] and URV is now being used as a quality indicator [14]. It has been previously pointed out that the best time frame to evaluate URVs was not defined [15–17]. However, their reduction is a quality objective [18, 19]. URV rates of 0.4% to 49.3% between 48 hours and 6 months have been reported [14, 15, 17], with the highest values for elderly subjects [13, 15, 17, 19, 20].

Various authors have proposed simple tools to evaluate the risk of readmission after a hospital stay using simple medico-administrative data [21]. To our knowledge, no surveys have been carried out to assess URV in the elderly after ED visits and the predictive value of readily available demographic and clinical variables in this setting. Similarly, very few studies have evaluated the clinical link between two ED visits, and some of these studies are quite dated. Moreover, it is indispensable to define more precisely the clinical link between two ED visits with an eye to defining populations with accrued risk of URV that could constitute subjects of intervention studies.

The aim of the present study was to give a clearer picture of URV and hospital readmission rates for elderly patients, and to determine predictive factors for URV and clinical relationships between two ED visits. We hypothesized that the time between two visits is an important criterion in the risk of URV and in the existence of a critical link between the two visits.

## Methods

### Study Design and Setting

This prospective observational study was carried out between January 1, 2010, and September 30, 2011 in a 1,000-bed university hospital located in the Paris urban area. The hospital has an Observation Unit (OU) and an acute geriatric unit, as well as medical and surgical wards (MSW).

### Study Population

All elderly patients seen in the ED were included. Even if +65 years old is currently accepted as elderly people by WHO, previous studies on interventions targeting the elderly population to reduce ED utilization in developed countries have been included patients aged greater than 55, 60, 65, 70 and 75 years old [22]. As 75 years old is the currently accepted value in France to define geriatrics patients, elderly population was defined as patients aged 75-years and over. Hospitalized and non-admitted patients after the index ED visit were included. The exclusion criteria for hospitalized patients were death during hospital stay and hospitalization in a palliative care unit.

### Data Collection and Measurements

Data were extracted from the computerized ED system (Urqual; McKesson). Easily available data on ED—demographics, patients’ characteristics, clinical data, care pathways and time intervals (minutes) currently used as ED quality indicators—were evaluated as predictive variables for URV and closely related clinical conditions.

The following variables were studied: age, gender, triage acuity level measured using a 5-point scale (level 1, resuscitation; level 2, emergency; level 3, urgent; level 4, less urgent; level 5, non-urgent). Care pathways were defined in the following manner: (i) ED→not-admitted, (ii) ED→admitted to MSW, (iii) ED→transfer, (iv) ED→OU→not-admitted, (v) ED→OU→admitted to MSW, (vi) ED→OU→transfer. Diagnostic categories were constructed on the basis of ED principal diagnosis (ICD-9) and categorized as (i) syncope, altered general status; (ii) neurological, pulmonary, internal medicine; (iii) falls, trauma, cardiovascular; and (iv) abdominal and urological. From the electronic medical records, the time of arrival and departure from the ED or OU were recorded as well as the departure date for hospitalized patients. For comparison purposes and as a function of observed URV rates, some categories were associated in Groups according to observed rates or clinically significant features. Time interval quality indicators were defined as follows: (i) ED length of stay; (LOS): time in minutes from ED arrival to the time the patient left the ED; (ii) EDOU-LOS: minutes between arrival at ED and the time the patient left the ED or the OU.

For each patient returning to the ED, we evaluated the relationship between the two visits. By reviewing the ED and hospital medical records, two investigators determined independently whether there was a link between the index visit and the second visit. In the event of disagreement, the files were reviewed by a third investigator for the final decision. To our knowledge, no definition had previously been proposed. Thus, we defined the relationship between ED visits as follows: (i) closely related clinical condition: the reason for the two ED visits is the same (principal complaints or diagnosis) or is the result of an unfavorable evolution or the secondary effects of the given treatment; (ii) probably related clinical condition: the reason for the second visit is not the same as that of the index visit, but the co-morbidity responsible for the two visits is the same; (iii) probably not related: the reason for consultation is different and is not related, even if the second visit is related to a known co-morbidity, but not the same responsible for the index visit; (iv) not related: new complaints not clearly related to known co-morbidities.

### Outcome Measures and Follow-up

All patients were followed 90 days after the index ED visit. URV rate was measured at 72 hours, 30 and 90 days after ED discharge or after hospital departure for hospitalized patients. Hospital readmission rate was defined as the number of patients readmitted to the hospital through the ED during the 90-day study period, and then adding those admitted directly to hospital MSW.

### Statistical Analysis

Univariate logistic regression analysis was used to study the association between demographics, clinical data, clinical care pathways and ED time interval quality indicators, and pre-defined end-points [23]. Variables that showed either a significant result or were near statistical significance (p<0.1) were included in the multivariate stepwise logistic regression model to determine the factors that were independently related to end-points. The models were built by using forward selection, and the p-value for entering and staying within the model was set at 0.05. Kaplan-Meier analyses were carried out in order to evaluate the relationship between time-to-return and explanatory variables. Free-of-URV time was estimated using the product-limit method. A survival analysis was carried out using the Gehan's or Wilcoxon's log-rank tests as indicated. To assess the independent influence of variables on outcome measures, Cox proportional hazard models were constructed. Model fit was determined calculating C-statistics to reflect overall fit [24]. Intra—reader and inter-reader agreements for clinical links between two visits were assessed by calculating a κ coefficient between the readers [25]. A two-tailed value of p<0.05 was considered statistically significant. All statistical analyses were carried out using Statistica 10 software.

### Ethical Approval

All datasets were completely anonymous and did not contain any identifiable personal health information. The dataset is currently being used by the ED as a quality and performance measure as part of an ongoing emergency activity and performance evaluation approved by the Assistance Publique-Hôpitaux de Paris committees on ethics, research and information. This study has been approved by the Emergency Ethics Committee by the Assistance Publique-Hôpitaux de Paris. Data anonymization was applied destroying tracks, electronic trail, on the data that would lead an eavesdropper to its origins. Participants provide their verbal informed consent to participate in this study after receiving structured information provided by ED medical staff members. Patients or family approval were obtained and recorded. Ethics committee approved this procedure.

## Results

### Main Characteristics of the Study Population

**Fig 1:**
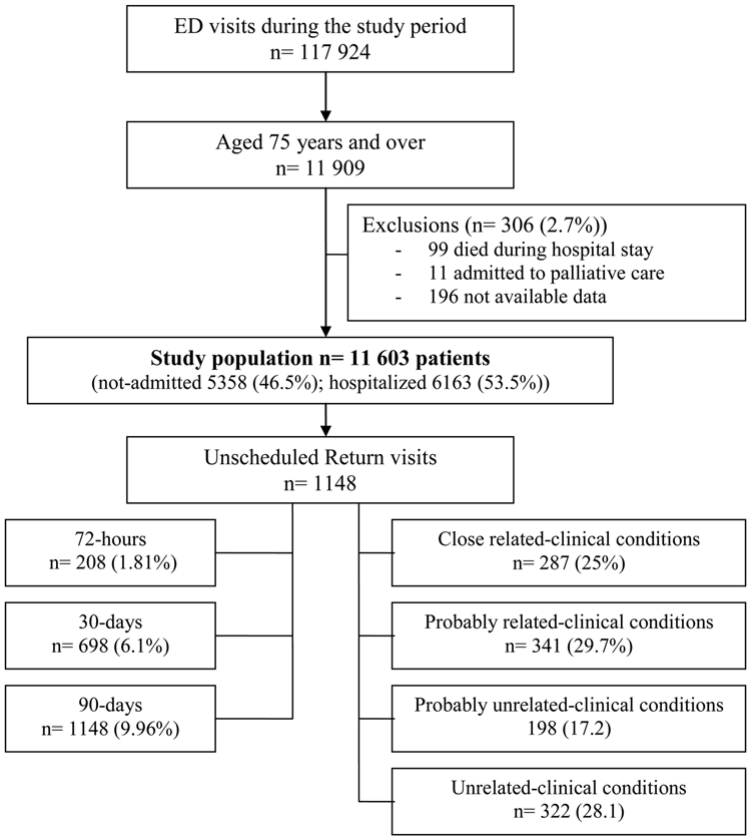
Flowchart of the study population.

Fig 1 details the study population flowchart. During the study period, 117,924 visits were registered by the ED. Among these, 11,603 patients (9.8%) were ≥75-years-old.

Table 1 shows the main characteristics of the study population. Overall admission rate (6,153/11,521 patients) during the first ED visit was 53.5% (admission to Hospital Geriatrics or MSW, or transfer).

**Table 1:** Demographic and clinical characteristics of the study population.

		number (%) or mean ± SD
Sex	Male / Female	4433 (38.5) / 7088 (61.5)
Age		84.1 ± 6.2
Age categories	<85 years	6305 (54.7)
	≥85 years	5216 (45.3)
Time of visit	Day (8 am to 8 pm)	8555 (74.3)
	Night (8 pm to 8 am)	2966 (25.7)
Diagnostic category	Syncope, altered general status	1616 (14)
	Neurological, pulmonary, internal medicine	4359 (37.8)
	Falls, trauma, cardiovascular	3951 (34.3)
	Abdominal, urological	1595 (13.8)
Triage / Acuity Groups	Levels 1 to 3	9785 (88.1)
	Levels 4 to 5	1319 (11.9)
Clinical care pathway	ED→ not-admitted	4156 (36.1)
	ED→ MSW	2039 (17.7)
	ED→ transfer	765 (6.6)
	ED→ OU→ not-admitted	1202 (10.4)
	ED→ OU→ MSW	1838 (16)
	ED→ OU→ transfer	1521 (13.2)
Final decision disposition	Transfer	2286 (19.8)
	MSW	3877 (33.7)
	Non-admitted	5358 (46.5)
For patients admitted to a MSW	Intensive care areas	195 (5)
	Internal medicine	708 (18.2)
	Geriatrics	437 (11.2)
	Surgical wards	1087 (28)
	Medical wards	1462 (37.6)
Time intervals (minutes)	Wait time to triage nurse	10.5 ± 9.4
	Wait time to ED provider	59.3 ± 43
	ED LOS (minutes)	228 ± 155
	ED plus OU LOS (minutes)	1051 ± 1625
Unscheduled Return Visits (URV)	72-hours	208 (1.8)
	30-days	698 (6.1)
	90-days	1148 (10)
Time interval (days) from ED visit to URV		28.7 ± 26.3
URV during follow-up periods	≥49 and 90 days	289 (25.2)
	≥21 and <49 days	279 (24.3)
	≥8 and <21 days	240 (20.9)
	<8 days	340 (29.6)
Final disposition decision for URV	Transfers	290 (25.3)
	MSW	86 (7.5)
	Non-admitted	772 (67.2)
For patients with URV	Unrelated clinical condition	322 (28.1)
	Probably unrelated clinical condition	198 (17.2)
	Probably related clinical condition	341 (29.7)
	Close related clinical condition	287 (25)

ED: Emergency Department; MSW: geriatric, medical or surgical ward; OU: Observation Unit; LOS: length of stay; URV: unscheduled return visits

#### URV and re-hospitalization rates

In total, 208 patients (1.81%), 698 patients (6.1%) and 1,148 patients (9.96%) returned to the ED within 72 hours, 30 and 90 days after the index ED visit, respectively. Median time to URV was 17 days.

Hospital admission rate during the second ED visit was 32.8% (376/1,148). Another 82 patients were admitted directly to hospital Geriatrics or MSW. Thus, 90-day hospital readmission rates were 3.3% (376/11,521 patients) for through-ED admissions and 4.0% (458/11,521) after adding direct hospital admissions.

### Predictors for URV

**Fig 2:**
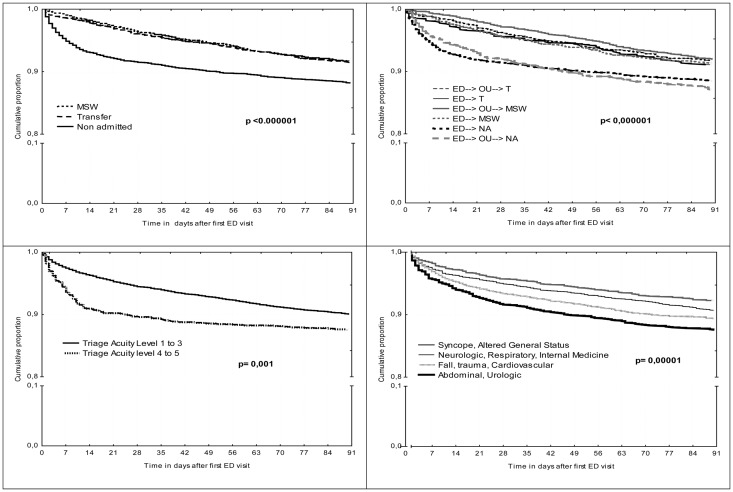
Kaplan-Meier curves showing Emergency Department (ED)-unscheduled return rate as a function of ED patient complexity evaluation.

In the overall study population, multivariate logistic regression models indicated that the ED final disposition decision (Transfer; MSW; Non-admission) was a significant 30-day URV predictor [OR: 1.52 (95% CI 1.42–1.56); 2.32 (2.12–2.38); p<0.0001]; and that Care pathways were significant 90-day URV predictors (ED→OU→MSW; ED→OU→transfer; ED→transfer; ED→MSW; ED→OU→non-admitted; ED→non-admitted) [OR 1.11 (95% CI 1.08–1.12); 1.23 (1.19–1.24); 1.35 (1.31–1.36); 1.50 (1.44–1.51); 1.66 (1.64–1.67); p<0.001]. Through time-to-failure analysis, and by observing URV curves, we determined that the most rapid returns concerned non-admitted patients. Fig 2 shows the time-to-failure curves for URV for variables with statistically significant results (P values indicate Log-Rank tests).

Then, we performed a time-to-failure analysis for predictive factors of 30-day URV in function of the final disposition decision: non-admission and hospitalized (geriatrics, MSW or transfer). Among non- admitted patients, Log-Rank tests were as follows: sex (p = 0.4), age groups (<85, ≥85) (p = 0.2), time of visit (day, night) (p = 0.1), ED-LOS (0.25), EDOU-LOS (p = 0.3), wait-time to ED physician (p = 0.9), triage level (p = 0.009), and diagnostic categories (p<0.001). Among those who were hospitalized, we found: sex (p = 0.06), age groups (<85, ≥85) (p = 0.8), time of visit (day, night) (p = 0.03), ED-LOS (0.7), EDOU-LOS (p = 0.04), wait-time to ED physician (p = 0.6), triage level (p = 0.06), and diagnostic categories (p = 0.02). Table 2 presents the results of multivariate Cox regression models for 30-day URV. C-statistics values were 0.59 (95% CI: 0.53–0.64) and 0.54 (95% CI: 0.45–0.61) for non-admitted patients and admitted patients respectively.

**Table 2:** Time-to-failure analysis for 30-day predictors for URV.

	Non admitted		Admitted*	
	OR (95%CI)	P	OR (95%CI)	P
Time of visit				<0.05
Day time (8 am to 8 pm)			1	
Night-time (8 pm to 8 am)			1.32 (1.19–1.47)	
Diagnostic category		<0.001		0.007
Syncope, altered general status	1		1	
Neurological, pulmonary, internal medicine	1.27 (1.21–1.34)		1.2 (1.12–1.26)	
Falls, trauma, cardiovascular	1.44 (1.37–1.51)		1.29 (1.22–1.39)	
Abdominal, urological	1.59 (1.52–1.67)		1.40 (1.32–1.48)	
Triage Acuity Groups		0.01		
Levels 1 to 3	1			
Levels 4 to 5	1.32 (1.21–1.45)			

Multivariate Cox model regression analysis.

* Admitted patients include transferred and admitted in MSW patients.

OR: Odds Ratio; CI: confidence interval; ED: Emergency Department; MSW

### Predictors for Closely Related-Clinical Conditions

Intra- and inter-reader agreements were found to be good: 0.82 (0.59-.98) and 0.75 (0.41–0.96) respectively.

**Fig 3:**
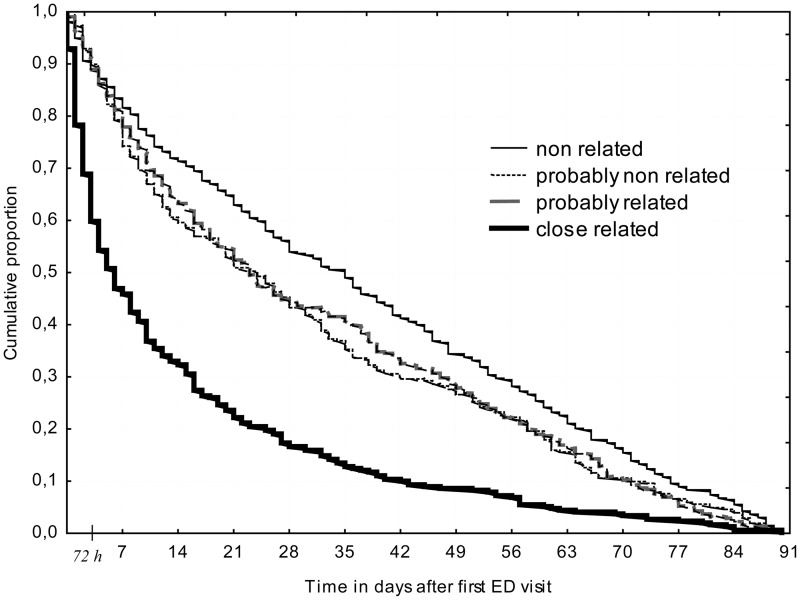
Kaplan-Meier curves showing Emergency Department (ED) unscheduled return rate as a function of clinical relationship between two ED-visits (n = 1148 patients).

Fig 3 shows the Kaplan-Meir curve (1,148 patients returning to ED) according to the clinical relationship category between the two visits.

Median times to URV were 5 days for closely related, 22 and 23 days respectively for probably related and probably non-related, and 35 days for non-related clinical conditions.

Table 3 shows the results of unadjusted and multivariate logistic regression analysis for associated factors to closely related clinical conditions (C-statistics = 0.77 [95%CI: 0.71–0.83]).

**Table 3:** Predictors of closely related clinical conditions (CRCC) between two ED-visits.

		Logistic regression analysis			
		Unadjusted		Multivariate	
		OR (95%CI)	P	OR (95%CI]	P
Sex	Female	1	0.6		
	Male	0.9 (0.7–1.2)			
Age	<85 years	1	0.5		
	≥85 years	0.9 (07–1.2)			
Time of visit	Day (8 am to 8 pm)	1	0.7		
	Night (8 pm to 8 am)	1.1 (0.8–1.4)			
Triage / Acuity Groups	Levels 4 to 5	1	0.08	1	<0.001
	Levels 1 to 3	1.43 (0.94–2.15)		2.66 (1.71–4.12)	
Wait time to ED Provider	<42 min	1	0.5		
	≥42 min	0.9 (0.7–1.2)			
ED LOS	<200 min	1	0.07		
	≥200 min	1.3 (1–1.7)			
EDOU LOS	<360 min	1	0.8		
	≥360 min	1 (0.8–1.4)			
Final decision disposition	Transfer	1	<0.001		
	MSW	1.74 (1.38–2.10)			
	Non-admitted	3.10 (2.34–3.57)			
Clinical care pathway	ED→ OU→ MSW	1	<0.001		
	ED→ MSW	1.18 (1.13–1.22)			
	ED→ transfer	1.40 (1.33–1.43)			
	ED→ OU→ non-admitted	1.66 (1.56–1.68)			
	ED→ OU→ transfer	1.96 (1.83–1.98)			
	ED→ non-admitted	2.32 (2.14–2.38)			

ED: emergency department; OU: observation unit; LOS: length of stay; MSW: geriatric or medical/surgical ward

## Discussion

In our study of URV after an ED visit from elderly patients, we determined that URV and hospitalization rates were within previously reported ranges [11–15, 19], that non admitted patients were at increased risk for URV, and that some factors were even significantly associated with URV in multivariate models while the overall prediction model value was low. Similarly, we found that closely related and probably related clinical conditions were frequent features, up to 50%, and that closely related clinical conditions occurred mostly during the first two weeks after ED or hospital discharge. Our study is the first to include patients aged 75 and over, non-admitted and hospitalized after an ED visit, as well as the first to evaluate the URV risk of admitted patients according to care pathways.

The evaluation of URV curves according to the final decision in emergency departments and care pathways clearly demonstrates that patients non-admitted through ED or OU have a heightened risk of URV and early URV. Although our hospital has an acute geriatrics unit, many geriatrics places are located outside our establishment, which could explain the protective character of transfers. Since it is currently accepted that hospitalizations degrade the autonomy of elderly patients [26] and that many hospitalizations are preventable [27], we have implemented strategies to reduce such hospitalizations. Our results do not question the general strategy of reducing admissions from the ED [28] and the development of alternatives to hospitalization. Rather, they indicate that a better definition of the criteria for hospitalization and optimal ambulatory care are required.

The Observation Unit (OU) has been proposed as the optimal evaluation tool for elderly patients in emergency departments [29]. In our department, geriatric evaluation is performed in the OU. However, passage through the OU does not modify the URV rate for patients non-admitted through ED or OU. We noticed that the hospitalization rate was higher for the first visit than during URV and resulted in few direct hospital hospitalizations. This seems to indicate that elderly subjects use EDs as a means of reevaluation or readmission to the hospital, indicating dysfunction in the care pathway as maximizing direct admissions and avoidance of EDs have been proposed as an approach to optimize healthcare and ED function [30].

We found in the overall study population that care pathway and final ED disposition decision were significantly associated with URV rate, indicating that non-admitted patients were at increased risk for URV. Then, we evaluated the factors associated with URV risk for non-admitted patients and hospitalized patients by using simple variables at the disposal of the emergency care provider. Two models for predicting risk of readmission after hospital discharge using easily available clinical and administrative data have been previously proposed [24–26], but only half of all discharged patients eventually readmitted were correctly identified [22, 31, 32]. The value of carrying out a geriatric clinical assessment (GCA) and various geriatric scores has been suggested; however most authors agree that this geriatric expertise takes too much time to be performed routinely in ED [31]. Our results confirm the globally low predictive value of these models for through-ED non-admitted and hospitalized patients alike.

Nevertheless, our study brings new elements to play thanks to its use of a time-to-failure analysis. Among non-admitted patients, low complexity clinical conditions, as defined by triage acuity scale, and diagnostic categories were associated with an increased risk for 30-day URV. It has been reported that errors in severity assessment are common among patients readmitted after hospital discharge [25]. Among admitted patients, we found that diagnostic categories and ED visit times (day or night) were significantly associated with 30-day URV. In our study, the night period was associated with a heightened URV risk for admitted patients after multivariate analysis, indicating that the context of the decision can impact the quality of the care pathway. Our results also indicate that—among non-admitted and hospitalized patients alike—some common geriatric conditions, i.e. syncope and altered general status, appear to be low risk clinical conditions, whereas neurological complaints including confusion and falls, and pulmonary and cardiovascular disorders, which are very common among geriatric patients in the ED, were significant predictors for 30-day URV.

Our objective was neither to assess the avoidable nature [33] nor the inappropriate use of ED visits [34], which are complex subjects, but rather to evaluate the clinical link existing between two ED visits. It has been previously reported that in the elderly, early URV is more likely to be related to the same unresolved problem [35, 36]. By using a simple and reproducible tool, we were able to obtain satisfactory inter- and intra-reader values for assessing the clinical relationship between two ED visits. We found that 25% of URVs were closely related clinical conditions and that up to 50% were closely related or probably related clinical conditions. We also learned that 50% and 80% of closely related clinical conditions returned within 5 and 21 days respectively, and that the time interval between index visit or hospital discharge and URV was the strongest predictor of closely related clinical conditions. Patients with the most acute clinical conditions (triage level 1 to 3) were at increased risk for CRCC. Our results indicate that early return visits—within the first three weeks—are those for which the original motivation for consultation or the evolution of the condition remains the main problem. Interestingly, care pathway does not appear to be related to the risk of closely related clinical conditions, indicating that a hospitalization decision does not reduce the risk of URV clinically related to the first visit.

The best time frame to evaluate URV was not defined [15]. We evaluated the impact of time lapse between first ED visit or hospital discharge and URV, and the relationship between the closely related clinical condition and time lapse between visits. We observed that a majority of URVs and most closely related clinical conditions occur during the first three or four weeks. Thus, we are able to estimate that the best time frame for evaluating URV and for studying the clinical relationship between two visits is 30 days.

ED-LOS is the most currently used ED quality indicator, and has been previously associated with patient morbidity and mortality [37]. Reducing the time a patient spends in the ED could result in an increased risk of URV [37], but this assertion remains controversial [13]. In the present study, short ED-LOS and EDOU-LOS were not found to be linked to a risk of URV, indicating that short visits to the ED or OU are unrelated to deterioration in the quality of care of elderly patients.

The present study has several limitations. First, patients were included in one single ED located in a densely populated urban area. Secondly, we did not evaluate the use of other healthcare resources such as consultations with a general practitioner; inappropriateness of post-ED disposition is associated with unscheduled returns to the ED [26]. Third, the risk of death during follow-up after the index visit is estimated at 15% [20]. We excluded patients who died during hospitalization and those admitted in palliative care units. Moreover, some patients may have died at home without returning to the ED or being admitted. Fourth, we did not evaluate returns to ED in other hospitals, however this risk was considered to be low as more than 95% of our elderly patients were living close to our hospital. Nevertheless, our study is the first of its kind to include patients, both admitted and non-admitted through ED, and to evaluate care pathways through ED as well as the existence of a clinical relationship between two visits. It is also the first to include the time factor in analyzing factors related to URV risk and closely related clinical conditions.

## Conclusions

Although our study confirms that models for identifying geriatric patients at increased risk for URV after ED or hospital discharge are of little value, we have characterized patterns of risk for URV, notably ED discharge even after OU stay, and some frequent clinical conditions in the elderly. Even if many of the identified risk factors for URV are not modifiable, our results indicate that URV can be used as a quality indicator, and that future studies to evaluate the impact of intervention measures to reduce URV rates must evaluate the clinical link between two ED visits—30 days after the first ED visit—and target rather homogenous population groups in light of the difficulty of defining sub-groups at heightened risk.
